# Reduction of Canine Plasminogen Leads to an Expanded Molecule which Precipitates

**DOI:** 10.1371/journal.pone.0006196

**Published:** 2009-07-10

**Authors:** Jack A. Kornblatt

**Affiliations:** Enzyme Research Group, Department of Biology, Concordia University, Montreal, Quebec, Canada; Griffith University, Australia

## Abstract

Canine plasminogen is made up of seven domains. In each domain there are several cysteines that are linked by disulfide bonds. Reduction of a limited number of the cystines destabilizes the protein such that it precipitates. The bond or bonds that are broken provide about 14 kcal of stabilization energy. Circular dichroism and dynamic light scattering indicate that there is probably an intermediate that is formed prior to precipitation and that the intermediate is somewhat larger than the compact form of plasminogen.

## Introduction

Plasminogen (accession number P00747) is a blood protein with multiple roles. It binds to many different chemical species; once bound, it can be activated via proteolysis to plasmin [1], [2]. The major role for plasminogen is clearing the circulatory system of blood clots [3]–[5]. Plasminogen is thought to play a key role in the tissue remodeling that occurs during pregnancy [6], [7] and that which occurs during the metastasis of tumors [8], [9]. Lastly, plasminogen is thought to help in the propagation of bacterial infections [10]–[12] as well as diseases associated with prions [13]–[15].

Human plasminogen is a 791 amino acid containing protein [16], [17]. Activation occurs by cleaving a single peptide bond, between residues 561 and 562 [18]–[20]. Activation can be rapid or slow, the rate depending on the activator and the conformation being acted upon. The activation process and its dependencies have been reviewed [3], [21], [22].

There is no complete three dimensional X-ray structure for plasminogen but the X-ray structures of six of the seven individual domains are known. The sequence of domains is: the N-terminal peptide followed by a series of five kringles followed by a proteolytic domain at the carboxy-terminus. All plasminogens have approximately the same structure. Canine plasminogen (DPGN), the protein used in this work, consists of 793 residues which include 24 disulfide bridged cystines (48 cysteines) [23]. There are multiple conformations of plasminogen and the protein populates the various forms. There are three classical forms which are dependent on ligand binding: two which are open [24], [25] and one which is closed; there are compact and relaxed structures which are the result of high and low temperatures [26]; there are truncated open structures which are the result of proteolysis of the N-terminal peptide. The conformation used in this study was the full length, native, closed structure referred to as Glu-plasminogen.

Many blood proteins are synthesized by the liver and subsequently exported. Blood proteins frequently contain disulfide bonds which result from the oxidation of cysteine residues that are closely positioned in the three dimensional structure. Oxidation does not usually take place in the cytosol of the cell in which synthesis has occurred because the cytosol of most cells is buffered at about −0.3 volts. In this reducing atmosphere, the majority of cysteines will exist as free sulfhydryls. Blood proteins and other exported proteins are oxidized as they leave the cytosol and enter the endoplasmic reticulum [27]. In the case of plasminogen, simultaneous export and oxidation must take place because the intact protein is not soluble in the reduced state. This has been shown to occur during synthesis of protein by HEPG2 cells [28], [29]; when synthesis occurs in the presence of dithiothreitol, there is formation of aggregates which contain several different protein species .This study shows that one or two of the 24 disulfide bridges in plasminogen are especially labile and, on reduction, expose groups which bring about precipitation of the protein.

## Results and Discussion

**Figure 1:**
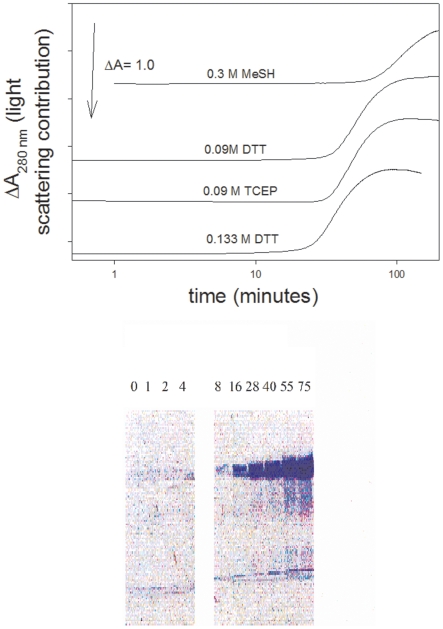
Reduction of 1.8 µM DPGN leads to precipitation. (Top panel) Plasminogen was treated with mercaptoethanol, dithiothreitol or TCEP at the concentrations shown in the figure. Precipitation was followed by monitoring the change in light scattering/Absorbance at 280 nm. The starting absorbance was about 0.4. Each curve has been offset for ease of viewing. The buffer was 5 mM K_2_HPO_4_, 5 mM KH_2_PO_4_, 100 mM NaCl, pH 7; the temperature was maintained at 20°C. (Bottom panel) At the times indicated in the figure, 0.1 mL of the sample containing 0.133 M dithiothreitol was removed from the cuvet and immediately centrifuged at 13 000 rpm. The precipitate and supernate were separated. SDS sample buffer was added to each. Both were subjected to SDS-PAGE on 7% gels. The precipitates are shown in the figure. The gels of the supernates were not sufficiently stained with Coomassie Brilliant Blue as to make the plasminogen easily visible.

Plasminogen was incubated with three reductants, mercaptoethanol, DTT (Cleland's reagent), or tris(2-carboxyethyl)phosphine (TCEP), and the change in light scattering monitored at 280 nm (Figure 1, top panel). Each of the four samples shown in Figure 1 (top) has had the signals offset to higher values so that the data can be easily seen. Figure 1 (top) shows that all three reductants gave rise to a large change in A_280_. The change is the result of precipitation of the protein. The time at which precipitation occurred was dependent on the reductant and its concentration. At higher concentrations, the precipitation occurred more rapidly.

Figure 1 (top panel), also illustrates the time dependency. The time at which the optical signal changed was 35 minutes when the DTT was 0.133 M compared to 60 minutes when it was 0.09 M. The mixture of 0.133 M DTT and plasminogen, shown in the top panel, was sampled and centrifuged at different times during the incubation. Each precipitate was dissolved in SDS and run on SDS-PAGE. The bottom panel of the figure shows the composition of the centrifuged material. In the 0–8 minute time frame there was no change in absorbance and no precipitate in the centrifuged material. After that there was a rapid increase in A_280_ and in the amount of precipitate on the gel. The major band migrated with authentic plasminogen. The experiment clearly showed that in the presence of DTT changes in absorbance and light scattering occurred at the same time as precipitation of the plasminogen. When plasminogen was reduced by mercaptoethanol or TCEP and the precipitate subjected to SDS-PAGE, similar results were obtained. As absorbance changes, plasminogen appears in the precipitate.

**Figure 2:**
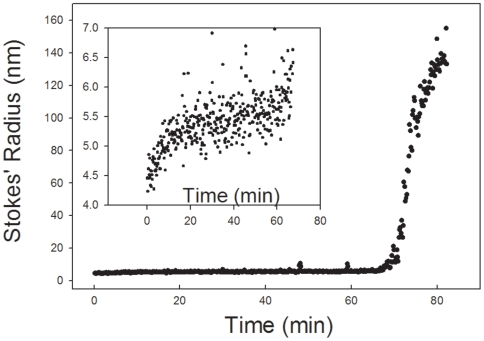
Dynamic light scattering of DPGN after addition of reductant. DPGN was mixed with TCEP, filtered through a 0.02 µm Whatman filter into a 20°C cuvet and the dynamic light scattering measurements started immediately. 2.4 µM DPGN, 41 mM (pH adjusted) TCEP, 170 mM KPi, 100 mM NaCl and 1 mM NaN_3_. The inset is an expanded version of the first 60 minutes of the reaction. K_2_HPO_4_ was used to adjust the pH of the TCEP.

Dynamic light scattering can be used to monitor the period between the onset of reduction and the formation of the precipitate (Figure 2). Dynamic light scattering measures light that is scattered from a tightly focused laser. The light scattered by the particles in solution can be related to the size of the particles and is dependent only on the diffusion coefficients of the particles and the viscosity of the medium containing the particles. In the main panel of Figure 2, extremely large particles started to appear at about minute 75 when plasminogen was reduced with 41 mM TCEP. The inset to the figure indicates that there was an intermediate particle that started to appear immediately in the presence of the reductant. The intermediate was maintained until there was major aggregation. In addition, whereas the Stokes' radius of native plasminogen in the absence of an opening ligand was close to 4 nm, the size of the intermediate was significantly larger than that of the native, closed, protein. The rate and persistence of the intermediate was dependent on the concentration of DPGN and reductant as well as the identity of the reductant.

**Figure 3:**
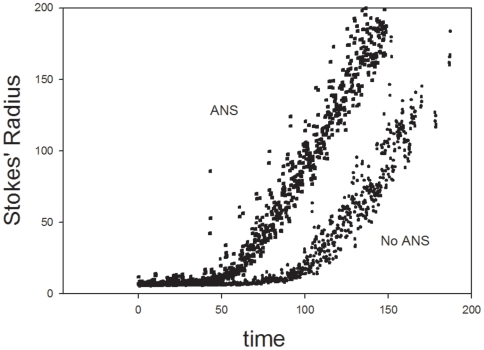
The influence of ANS on the dynamic light scattering of 1 µM DPGN in the presence of 10 mM (pH adjusted) TCEP. The solid squares are in the presence of 20 µM ANS whereas the solid circles are in its absence.

The presence an intermediate(s) whose size is somewhat larger than that of the native species may be indicative of a molten globule [30] type state or states between the native and the precipitate. The molten globule is postulated to have an expanded core relative to the native protein resulting in a somewhat larger molecule with a greater Stokes' radius (see Figure 2, inset). In addition, the molten globule is postulated to expose hydrophobic surfaces that had been sequestered in the native protein. ANS [31]–[33] has been used as a probe of newly exposed hydrophobic surfaces and molten globules; it intercalates between exposed hydrophobic surfaces. The view is that these newly exposed, hydrophobic surfaces are sequestered from solvent in the majority of native proteins but some become exposed when the protein is induced to form a molten globule. The dynamic light scattering data of Figure 3 show that ANS and reductant acted synergistically to induce precipitation. The solid squares are the data in the presence of ANS while the solid circles are the data in its absence. The half time for the precipitation in the presence of ANS was about half that in its absence. ANS, by binding to the newly exposed hydrophobic sites, displaced the equilibrium between the native form of plasminogen and the expanded intermediate; the faster the expanded intermediate increased in concentration, the faster precipitation occurred. Is it critical whether the intermediate which forms on addition of reductant is a molten globule or some other expanded form? What is important is that there is an intermediate. That it may have characteristics of a molten globule-like structure is suggested by the data on size, ANS enhancement and finally by circular dichroism.

**Figure 4:**
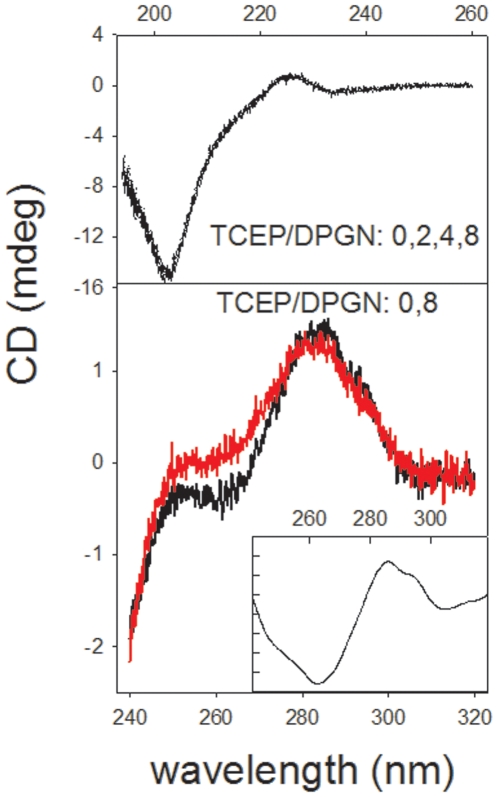
The influence of TCEP on the circular dichroic spectrum of DPGN. The top panel shows that the far UV spectra down to 190 nm is not influenced by TCEP at ratios of TCEP to DPGN of 0, 2, 4 or 8. The protein concentration was 3.2 µM in the 0 TCEP sample and 2 µM in the 16 µM TCEP sample. At these concentrations, the plasminogen did not precipitate. The bottom panel shows that the near UV spectrum of DPGN is disrupted by the TCEP; this region of the spectrum is characteristic of the aromatic amino acid residues as well as the disulfides. The concentration of plasminogen in the 0 TCEP solution was 11.2 µM; in the 75 µM TCEP sample, the plasminogen concentration was 9.4 µM. At these concentrations, the protein did not precipitate during the course of the measurements. In both panels, the blanks were subtracted from the spectra and the resultant spectra normalized for protein concentration. The inset of the bottom panel shows the difference spectrum (0 TCEP - 8x TCEP) in the near UV. The difference spectrum has been smoothed whereas the spectra in the two main panels have not been smoothed. The difference spectrum is dominated by a characteristic tryptophan spectrum but must also contain a contribution from at least one disulfide.

Circular dichroism can monitor protein secondary structure as well as the disposition of aromatic groups and cystine. The signature region for secondary structure is the far UV portion of the dichroic spectrum (240 nm–180 nm). In the region of the spectrum from 300 nm to 240 nm, the dichroic spectrum reports on aromatics that are in an asymmetric environment and on the cystines. When the far UV spectrum remains constant after a change in conditions but the near UV changes, it frequently indicates that one form of the probed molecule is molten globule like compared to the other [30]. Figure 4 shows that the far UV CD spectrum of plasminogen (top panel) was not influenced by reduction; the spectra were essentially the same as previously reported [34]. There are four curves in the top panel of Figure 4 and they superimpose. This indicates that the sum of the secondary structure of the molecule stays more or less constant. Native, full length plasminogen has an unusual far-UV CD spectrum as can be seen from the figure. It is quite different from the spectra of those proteins which consist primarily of alpha helix and beta sheet. The spectrum of the protein is dominated the five kringles which are very tight units, each tied together by three disulfides. They contain neither beta sheet nor alpha helix but have a well defined secondary structure that gives the characteristic sharp minimum shown at about 203 nm.

By contrast, the bottom panel shows that reduction does influence the near UV CD spectrum. This region results from the spectra of the three aromatic amino acids with a smaller contribution from disulfide bonds. The aromatics are not optically active themselves in the near UV but, when held rigidly in an asymmetric environment, exhibit optical activity [35]. The disulfide bond is inherently asymmetrical and exhibits optical activity. Figure 4, the bottom panel, shows a slight loss of the CD signal in the near UV. The difference spectrum shown in the inset (0 TCEP–8× TCEP) demonstrates that there is a major change in the tryptophan spectrum. Since at least one disulfide is also reduced, there must also be a change in the disulfide spectrum. Loss of a portion of the tryptophan signal is probably the result of loss of a selected asymmetric environment occurring near the disulfide(s) that are reduced.

**Figure 5:**
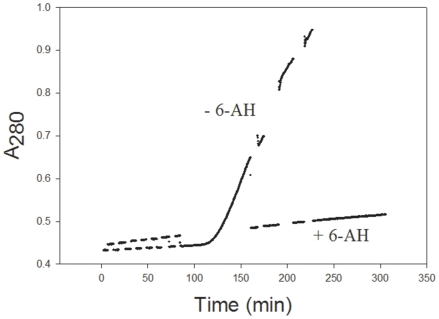
6-aminohexanoic acid inhibits the precipitation reaction. DPGN (2.3 µM) was treated with 15 mM dithiothreitol in the presence or absence of 26 mM 6-aminohexanoate. The buffer conditions were the same as in Figure 1.

One of the compounds that binds to plasminogen is 6-aminohexanoate (6-AH) (reviewed in [36]). When it binds it causes the plasminogen to open and change its hydrodynamic properties [24], [37]. The 6-AH can bind to four (1,2,4,5) of the five kringles, each of which has a different binding coefficient [38]; it is only on binding to kringle 5 that the protein opens [39]. While ANS enhances the rate at which the plasminogen precipitates, 6-AH inhibits the precipitation reaction as shown in Figure 5. The inhibition is specific. Glycine, which is a zwitterionic homologue of 6-AH and which similarly influences the dielectric coefficient of the medium, does not inhibit the precipitation reaction. Can this information be used as a guide to indicate which disulfide reductions are responsible for aggregation?

The structures of the kringles and the catalytic domain of plasminogen are well established [40]–[43]. There are a total of 24 disulfides in the intact plasminogen of which 16 are in the kringles, with three per kringle and one between kringles 2 and 3. Kringles 1, 2, 4, and 5 bind 6-AH and contain tryptophan residues which are quite near (3.2 Å) the disulfides. Disruption of any of the 12 disulfides which are in the 6-AH-binding kringle domains could lead to changes in the CD signal due to tryptophan. Other possibilities cannot be ruled out: (1) The bridge between kringles two and three is the disulfide bridge the reduction of which leads to precipitation. (2) 6-AH acts indirectly by causing the DPGN to open and it is only the closed protein which will precipitate. The disulfides in the kringles are most likely those that are responsible for the behavior of plasminogen following reduction.

How many disulfides are involved in the steps leading to precipitation? Mercaptoethanol and dithiothreitol (DTT) are poorly suited to answering this question. TCEP is a strong reductant with significant specificity for disulfides [44]. It was used in this study to count disulfides since it is not easily oxidized by molecular oxygen [44]. At high concentrations of DPGN and TCEP (>25 µM), precipitation was rapid and complete at ratios of 3 TCEP:1 DPGN (data not shown). This probably indicates that fewer than three disulfides need to be reduced prior to forming the species that precipitates. Mass spectrometry was used, unsuccessfully, to try to deduce which disulfides were reduced under these conditions.

**Figure 6:**
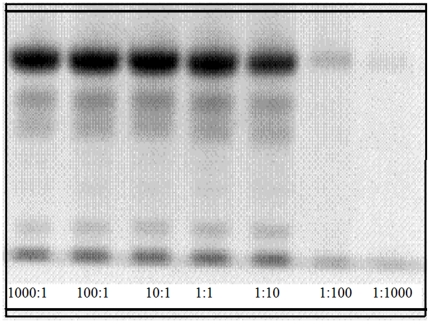
Precipitation of plasminogen by DTT/Dihydroxydithiane anaerobic mixtures. From left to right, the ratios are 1000∶1, 100∶1, 10∶1, 1∶1, 1∶10, 1∶100, 1∶1000. At 1∶10, about 50% of the plasminogen precipitates. E' (as an oxidation potential) of the mixture at this ratio is about 0.3 V. This gives a driving force of about 14 kcal for reduction of the plasminogen.

DPGN is extremely stable at room temperature in most buffers as long as there is no major activation of the protein to plasmin. Oxygen has no deleterious effects that can be easily measured. This stability ensures that in the oxidizing atmosphere of the blood, the molecule is not rapidly degraded. The fact that reductants can bring about a destabilization of the protein affords an opportunity to ask just how much stabilization energy is provided by the disulfide bridges. The question can be answered by setting the midpoint potential of the system, DPGN/reductant, such that the system goes to equilibrium rather than completion. The redox couple dithiothreitol/trans-dihydroxydithiane has a midpoint reduction potential, E'°, of −0.33 V [45]. *This value is approximately the same as the reduction potential found inside the cell*. In an anaerobic atmosphere there is no tendency for the DTT to undergo air oxidation. The mixtures of DPGN, DTT and dihydroxydithiane were prepared in a glove box in which the oxygen concentration was maintained at less than 5 ppm. The comparable concentration in air is 20% or 200 000 ppm. The samples were incubated for 72 hours at 25°C in open Eppendorf centrifuge tubes in the glove box. At the end of the incubation period, the samples were capped while still in the glove box, then removed and immediately centrifuged at 13 000 rpm for 10 minutes. The precipitates and supernates were separated. The protein in the precipitates was monitored by SDS-PAGE. The results are shown in Figure 6. The importance of the data in this figure is the following: At ratios of DTT/Dihydroxydithiane that are above 1∶10, the protein precipitates. At ratios below 1∶10, the protein does not precipitate. At 1∶10 DTT/Dihydroxydithiane about 50% of the protein has precipitated. At this ratio, the concentration of DTT is still 5 mM, well in excess of the plasminogen. The driving force for the reaction is given by the Nernst equation, expressed as an oxidation potential; it evaluates to 0.3 volts or about 14 kcal.
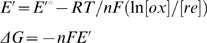



The conclusion drawn from this calculation is: the disulfides of plasminogen are functional. They stabilize the protein; the most labile of the 24 disulfides provide about 14 kcal of stabilization energy.

There are two aspects of the work that is reported here that are of general interest:

1. Mammals are regularly subjected to infection by bacteria, are constantly undergoing tissue remodeling, and are under constant threat from both internal and external sources. That they do not die every time a blood clot becomes lodged in a small artery is at least in part because plasminogen recognizes the insult. Infection with a pathogenic form of streptococci or a “scrapie” form of a prion requires tight regulation of plasminogen activation in order to control the infection. Cell debris from clots, as well as plasminogen-bacterial complexes and prion-plasminogen complexes can gain access to cell interiors. In the blood and in the intercellular space, the complexes are in rapid solution-equilibrium. Once brought into the cell, regardless of the mechanism by which entry occurs, the complexes are subjected to reducing conditions. Plasminogen which is tightly bound to its ligands is probably degraded at the same time as the ligand. Plasminogen which has been internalized in the absence of ligand poses a threat to the cell since it can now precipitate and thereby induce yet another potential insult. It's clear that this process must be controlled and it will be interesting to determine just how it is controlled.

2. Reductants induce precipitation without exerting a *large* influence on the secondary or tertiary structure of plasminogen. Precipitation is not a prerequisite for crystallization of proteins but frequently provides a guide for determining the solubility of the protein. We have carried out crystallization screens with canine plasminogen and reductants. The screens have indicated some formation of crystals. The best crystals have been grown in solutions containing PEG 400, ammonium sulfate, HEPES and a 2× excess of TCEP. So far the crystals have not been of a quality that is appropriate for X-ray analysis. It is hoped that those who work with the plasminogens from other species may have more success.

In conclusion, the data presented in this report indicate that the disulfides of plasminogen are necessary for maintaining the high concentration, *ca* 2 µM, of plasminogen in plasma. As the disulfides are reduced, hydrophobic surfaces must become somewhat more exposed thereby reducing the solubility of the protein, i.e., causing it to precipitate.

## Materials and Methods

The blood used for the preparation of plasminogen was obtained from the Montreal General Hospital, in 2005, following experimental surgery on dogs. The surgery had been approved by the hospital ethics board. The blood was treated immediately with sodium citrate such that the final concentration was about 15 mM. The resulting plasma was “straw” colored and showed no evidence of haemolysis. The preparation of plasminogen from plasma has been extensively described [5]. After the final lysine sepharose column, the protein was dialyzed against 10 mM potassium phosphate, 100 mM NaCl, pH 7.0. 0.3 µM Aprotinin (Fluka, St. Louis, MO) was added to the preparation at the time it eluted from the lysine-sepharose column. The salts used in this study were of ACS grade or better. 6-aminohexanoate (6-AH), 8-anilinonaphthalene sulfonic acid (ANS), mercaptoethanol (MeSH), Cleland's reagent (dithiothreitol, DTT), trans-4,6-dihydroxy-1,2-dithiane were from Fluka or Sigma (St. Louis, MO).

The actual precipitation of plasminogen in the presence of reductant was followed either with a Cary 2400 UV-VIS-NIR spectrophotometer (Varian Instruments, Toronto, CA) at 280 nm or with a Dyna-Pro dynamic light scattering apparatus (Wyatt Instruments, Santa Barbara, CA). Both instruments were thermostated, usually at 20°C. Samples were filtered through Anotop 10, 0.02 µm inorganic membrane filters (Fisher Scientific Co., Montreal, QC) prior to a measurement. Circular dichroic spectra were obtained with a Jasco 815 polarimeter thermostated at 20°C. The far UV spectra did not differ from those taken in the past [34]. SDS-PAGE, on the precipitated plasminogen, was carried out as previously described [46], [47] using 7% gels. The supernates were quite dilute (≪0.2 mg/mL) and were not monitored on gels.
